# Giving a voice to “the silent killer”: a knowledge, attitude and practice study of diabetes among French Guiana’s Parikweneh people

**DOI:** 10.1186/s13002-024-00713-9

**Published:** 2024-09-05

**Authors:** Michael Rapinski, Alain Cuerrier, Damien Davy

**Affiliations:** 1 Institut de Recherche en Biologie Végétale (IRBV), Université de Montréal, Jardin Botanique de Montréal, 4101 Sherbrooke Est, Montréal, QC H1X 2B2 Canada; 2 Laboratoire Écologie, Évolution, Interactions des Systèmes Amazoniens (LEEISA), CNRS, IFREMER, Université de Guyane, 97300 Cayenne, French Guiana France

**Keywords:** Diabetes, Palikur, French Guiana, Ethnomedicine, Ethnobiology, Knowledge, Attitude, Practice, Food, Medicine

## Abstract

**Background:**

The prevalence of type 2 diabetes (T2D) in the French overseas department of French Guiana, South America, nearly doubles that in its European counterpart, Metropolitan France. This region is demographically diverse and includes several populations of Indigenous Peoples. Although such populations are at particular risk of developing T2D across the Americas, very little is known about their health status in French Guiana, and accurate numbers of diabetic patients do not exist.

**Methods:**

In light of a potential public health crisis, an ethnomedicinal study of diabetes experienced by Indigenous Parikweneh was conducted to provide better insight into the knowledge, attitudes and practices (KAP) related to this quickly emerging disease in French Guiana. Altogether, 75 interviews were conducted with community members and Elders, as well as healthcare professionals and administrators providing services to the Parikweneh population of Macouria and Saint-Georges de l’Oyapock.

**Results:**

Interviews suggest a high incidence of T2D in this population, with cases that have risen quickly since the mid-twentieth century. Parikweneh participants linked the development of the illness to dietary changes, notably through the introduction of new and sweet foods. Recognizing the complexity of diabetes and its symptoms, diabetic patients highlighted the importance of biomedical treatments and follow-ups, though they frequently alternated or used them concomitantly with Parikweneh medicines. With the help of biomedical tools (i.e. glucometer), local medicinal practices mirrored biomedical approaches through dietary adaptation and the use of medicinal animals and plants for glycaemic control and the treatment of complications from the disease.

**Conclusion:**

Parikweneh are appropriating T2D into their knowledge system and adapting their health system in response to this relatively new health concern. A greater understanding of local practices and perceptions relating to T2D among medical staff may therefore be beneficial for meeting patients’ needs, providing greater autonomy in their health path, and improving treatment outcomes.

## Introduction

**Fig. 1:**
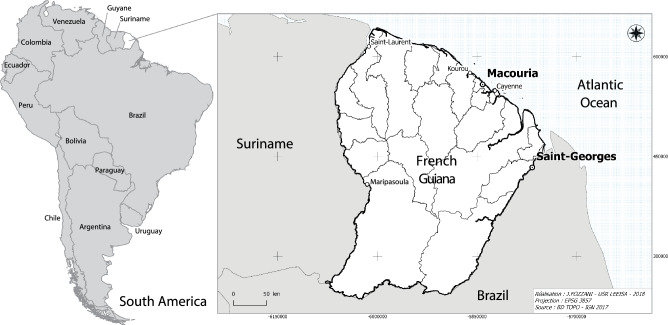
Locations of Parikweneh villages in French Guiana, namely in the communes of Macouria (5° 00′ N, 52° 28′ W) and Saint-Georges de l’Oyapock (3° 53′ N, 51° 48′ W). Figure reproduced and adapted from Rapinski et al. [18]

The number of people pharmacologically treated for diabetes nearly doubled in France from 2000 to 2015, from 2.6 to 5.0% of the population, affecting nearly 3.3 million individuals [1, 2]. The prevalence of diabetes, however, is notably higher in French overseas departments, particularly in French Guiana, where 8.08% of the population, age-adjusted, was estimated to be diabetic in 2015, a rate that has more than doubled within a 10-year span [2–5]. With a rate of type 2 diabetes (T2D) estimated at 8.12% by the International Diabetes Federation, French Guiana placed itself among South and Central America’s 10 regions with the highest prevalence of diabetes in 2013, just below Brazil [6, 7], which borders the French department (Fig. 1).

The demographic makeup of French Guiana is characterized by a diverse array of Creole, Maroon and Indigenous cultural groups. This diversity stems from significant movement and migration from Suriname, Brazil and Haiti [8]. Similar to other regions in the Americas where Afro-Descendants and Indigenous Peoples experienced colonization, these populations face a heightened risk of developing diabetes [9–16]. Factors contributing to elevated rates include lifestyle changes (e.g. food and nutrition transitions, sedentarization); potential genetic predispositions; and historical, political and psychosocial influences, which are all indicators of the impact of colonial history on family values and societal structures, as well as cultural and spiritual practices [1, 3, 5, 17].

Epidemiological statistics on Creole, Maroon and Indigenous populations are not available in French Guiana due to French legislation preventing ethnic discrimination in national studies. It is therefore difficult to make appropriate comments on the issue of diabetes in these cultural groups at risk, but data suggest that some of them are more affected than others. Medical dispensaries, locally called Delocalized Centers for Prevention and Care (*Centres Délocalisés de Prévention et de Soins;* CDPS), of the regional Hospital Center Andrée Rosemon (*Centre Hospitalier de Cayenne Andrée Rosemon;* CHC) in Cayenne report the largest numbers of diabetic patients in Saint-Georges de l’Oyapock and Maripasoula [19], municipalities characterized by high populations of Parikweneh, also named Palikur (Indigenous group), and Aluku (Maroon group), respectively. Nevertheless, an overall look at French Guiana suffices for various epidemiological studies of diabetes in France to associate its exceptional rates with the peculiarities of geographic isolation and indices of socioeconomic performance, all of which tend to vary along cultural grounds [2, 20–22].

Nimuendajú [23, 24] and Fernandes [25] reported relatively good health status among the Parikweneh from 1925 to 1950, although by 1978, the first reports of diabetes were recorded among those living in Brazil [26]. Since the sixteenth century, Parikweneh have maintained contact with European settlers and the Western world; however, like many colonized groups, the pace of lifestyle changes has accelerated since the mid-1960s [27]. Changes relating to lifestyles include evangelization, monetization, participation in the wage economy, sedentarization, industrialization, schooling and dietary changes, especially since 2003 with the opening of the region along the Brazilian border through road access [28–31]. These changes in lifestyle have resulted in an increasing dependence on commercially available and processed foods (i.e. sugar, powdered milk, coffee, condiments, oil, cookies, rice, butter, bread and soft drinks). On the other hand, this shift has been detrimental to traditional food acquisition activities such as hunting, fishing and swidden-fallow cultivation of cassava (*Manihot esculenta* Crantz; Euphorbiaceae) [26, 29, 30, 30–33], which continues to be a dietary staple [18, 28].

In light of growing concerns about an emerging public health crisis, an ethnomedicinal study of diabetes experienced by French Guiana’s Parikweneh population was conducted, thus applying methods of ethnobiology and medical anthropology to provide better insight into the knowledge, attitudes and practices (KAP) related to this quickly emerging illness. The primary objective of this study was to document KAP related to diabetes from an emic perspective (i.e. from the point of view of the Parikweneh people). Tangentially to documenting knowledge regarding diabetes, the perceived causes and factors involved in the development of this illness were compared to those expressed by workers associated with the healthcare sector to assess how these align with the biomedical perspective.

## Materials and methods

### Ethnographic background

Known as Palikur in French and English, the self-designated Parikweneh or Pahikwene speak the Parikwaki language, classified within  the Maipuran subfamily of the Arawak language family [28]. During the nineteenth century, the majority of Parikweneh people resided along the banks of the Rio Urucauá, situated in the present-day Amapá state of Brazil, although oral history suggests a small presence on both sides of the Oyapock River, now forming the border between France and Brazil [34]. The establishment of the current Parikweneh settlements in French Guiana began after the territory was ceded to Brazil in 1900, with significant migrations dating back to the 1960s, marked by the creation of the village of Espérance I in Saint-Georges de l’Oyapock [30], followed by the village of Kamuyene in Macouria. Owing to French legislation that prohibits the recording of census data distinguishing ethnicity, precise figures regarding the Parikweneh population in French Guiana are unavailable, nor are the prevalence of illnesses such as diabetes. However, self-reported estimates from Parikweneh leaders in Saint-Georges de l’Oyapock and Macouria suggest approximately 1800 Parikweneh in French Guiana in 2018 [18], doubling the estimated number of 850 in 2001 [28].

This study encompasses the villages of Kamuyene and Norino in the commune of Macouria, as well as the villages of Espérance I and Espérance II in the commune of Saint-Georges de l’Oyapock (Fig. 1). Due to their size and proximity, residents of each village are identified in connection with their commune (e.g. the Parikweneh of Macouria and the Parikweneh of Saint-Georges). The languages spoken in these communities include Parikwaki, French Guianese Creole, French and Portuguese.

### Biomedical systems

Public and private biomedical care is available to Parikweneh villages in both the communes of Macouria and Saint-Georges de l’Oyapock. Parikweneh individuals holding French papers have access to French social services and assistance as well as a publicly funded and free medical infrastructure [28, 30]. Although national laws apply concerning the rights of migrants and undocumented individuals to health coverage, various factors—such as administrative, legal, and societal barriers—hinder access to healthcare services [35]. These challenges are especially pertinent to the Parikweneh from Brazil residing in French Guiana. In Saint-Georges de l’Oyapock, both public and private healthcare systems are within walking distances from most households. At the time of the study, a public health centre, or dispensary, with two doctors and eight nurses, operated alongside a private clinic, managed by one doctor, and a private practice for home care nursing, managed by three nurses. The publicly operated dispensary, or CDPS, operates as a delocalized pole for the health and prevention of the CHC, a hospital in Cayenne where patients are sent for surgery and emergency treatment.

In Macouria, healthcare services are within a short drive’s reach. The CHC is the closest public healthcare centre available to the Parikweneh of Macouria. Doctors, however, operate private practices in nearby towns and villages, namely two in Macouria and one in Cayenne that are attended by Parikweneh, along with a private homecare nursing service. The access and use of biomedicine are well integrated into the contemporary Parikweneh way of life. However, local medicinal practices remain well documented [36, 37].

### Consultations

Fieldwork spanned from June 2017 to July 2018. The distribution of interviews is detailed in Table 1. Conversations took place in the participants’ preferred languages, which included French and French Guianese Creole, as well as Parikwaki, with the assistance of an interpreter. Participants were selected based on their affiliation with one of four demographic categories and identified through snowball sampling. Initial names were provided through pre-existing relationships between community members and one of the authors (DD), who has been collaborating with Parikweneh communities for more than 20 years [38–41]. Additional names were suggested by consulting with local *Chefs coutumiers* (i.e. village Chiefs).

**Table 1:** Interview breakdown by communities and description of participant categories

Community	# of interviews	# of participants	Mean age (± SD)	Sex ratio (men/women)
Saint-Georges	32	43	53.4 (± 15.8)	23/20
Macouria	28	42	51.1 (± 15.4)	20/22
Cayenne*	15	16	42.3 (± 10.6)	4/12
Total	75	101	50.4 (± 15.2)	47/54

Category	Example
Community members and healthcare users	
Knowledge holders (KH)	Elders, healers
Healthcare users (HU)	All other community members
Healthcare network staff	
Healthcare administrators (HA)	Directors, administrators
Healthcare professionals (HP)	Doctors, nurses, nutritionists

Table reproduced from Rapinski et al. [18]

*Interviews conducted with participants in or with the healthcare sector

### Data collection

Information was gathered through semi-structured interviews and participant observation in community activities. The interview questions delved into individuals’ knowledge and understanding of diabetes and medicine, along with their attitudes and practices concerning both local and biomedical systems. Names, terms and related concepts were documented and recorded in Parikwaki. As several writing systems have been devised over time, vocabulary was transcribed according to the system developed by Green et al. [42], which was favoured by participants from Macouria and Saint-Georges. The spelling of words was verified by participants known for their literacy of the Parikwaki language and cross-referenced with the Parikwaki-Portuguese dictionary [42]; preferences were given to spelling provided by participants when they differed from the dictionary. Terminology, nomenclature and various other associated concepts were confirmed and validated during subsequent trips to the communities in 2019 and 2022.

### Data analyses

The interviews were transcribed verbatim, and the transcripts were then stored and processed in NVivo qualitative data analysis software [43] to identify, manage, link and retrieve the coded data. Participants who preferred to be interviewed together were considered *n* = 1, reducing the participant sample size. Finally, verbatim quotes from participants, translated into English by the authors, were used to further elucidate local concepts relevant to health and diabetes. Thematic analysis and sequential focused coding were used to identify key themes, terms and concepts from the interview transcripts pertaining to each major aspect of the KAP study.

#### KAP

Upon review, interview transcripts were first analysed to elucidate (1) knowledge, (2) attitudes and (3) practices associated with diabetes, based on the approach described by Odonne et al. [44].

1. *Knowledge of disease and treatments* Characterization of Parikweneh knowledge around diabetes focused on parts of the discussions pertaining to the naming of the disease, its definition, past and present, perceived symptoms, its origin and causes and knowledge of treatments, both biomedical and Parikweneh.
2. *Attitudes towards disease and treatments* Characterization of attitudes focused on parts of discussions that pertained to the therapeutic path taken by participants in the case of diabetes.
3. *Healing practices* Characterization of healing practices focused on parts of discussions that pertained to therapeutic approaches adopted by participants to treat and manage diabetes.

#### Quantitative data transformation and statistical analysis

The relative frequency of citations (FC), expressed as a percentage, was used to express the number of participants or interviews that discussed or referred to specific themes, terms or concepts. The causes and factors involved in the development of diabetes reported by Parikweneh participants and workers associated with the healthcare sector were compared by using Spearman’s rank correlation to determine if these factors were correlated. This nonparametric univariate statistical analysis was performed in IBM SPSS [45]. The significance of Spearman’s correlation coefficient (ρ) was tested at a threshold of *α* = 0.05 [46].

### Specimen collection

Application of the Nagoya Protocol in French law has applied since July 1, 2017. However, the administrative procedures for requesting authorization for research on traditional knowledge associated with genetic resources were not put in place in French Guiana until the end of 2019 [47], prior to the beginning of the COVID pandemic. This occurred after obtaining consent from participating communities to conduct research and the beginning of these activities. In the absence of administrative procedures to obtain national consent for the collection of genetic resources and associated traditional knowledge, no specimens could be collected for an in-depth ethnobiological investigation of the practice component of the KAP study. Species well known to food systems around the world, thus not concerned by the Nagoya Protocol, and documented in the scientific literature are presented in the results. Nonetheless, species names and identification that participants divulged throughout this study, for the most part, have not been included to comply with French legislation. This relates to species used for medicinal purposes or that have not previously been reported in the scientific literature concerning the Parikweneh food system. A more in-depth study of therapeutic practices developed by Parikweneh to manage diabetes will be conducted upon approval by the French system on Access and Benefit Sharing.

## Results

Overall, 75 interviews were conducted involving 101 participants, with a sex ratio (men/women) of 0.87 (Table 1). This included 28 non-Parikweneh (27 interviews) participants who were all professionally linked to the healthcare sector and 73 Parikweneh participants (48 interviews). Two Parikweneh participants were professionally linked to the healthcare sector at the time of fieldwork, whereas another, now retired, had been in the past.

### Knowledge of the disease

All 101 participants interviewed were familiar with diabetes. Among Parikweneh participants, 42.5% (31/73) of individuals, spread across more than half of the interviews (56.3%; 27/48), reported having diabetes. Only 2.74% (2/73) of respondents claimed that there was no diabetes in their immediate household (i.e. spouse, child or parent), including themselves. The remainder had at least one family member with diabetes, and one participant stated that she was no longer diabetic. Indeed, participants specified that the disease was particularly common, being one of the major health problems in their communities, along with cancer, high blood pressure and cardiovascular diseases. Participants reported a rise in the number of diabetic individuals, that increasingly younger people were diagnosed with diabetes and that this illness historically killed many community members.

#### Naming and definition

Regardless of the language of communication, Parikweneh participants most frequently referred to diabetes by the name *diabet* (100%)*,* a contemporary borrowing of the French and Portuguese words *diabète* and *diabetes*, respectively. However, the terms *sugku* (sugar) and *karayt sugku* (sugar disease) were used in 50% (24/48) of the interviews. One elderly couple specified that Elders used to call it *mbeyevye karayt *(Elders’ disease) because it used to primarily affect elderly people. Another participant called it *karayt masarahatya*, which translates to “disease that causes one to become dry”.

**Fig. 2:**
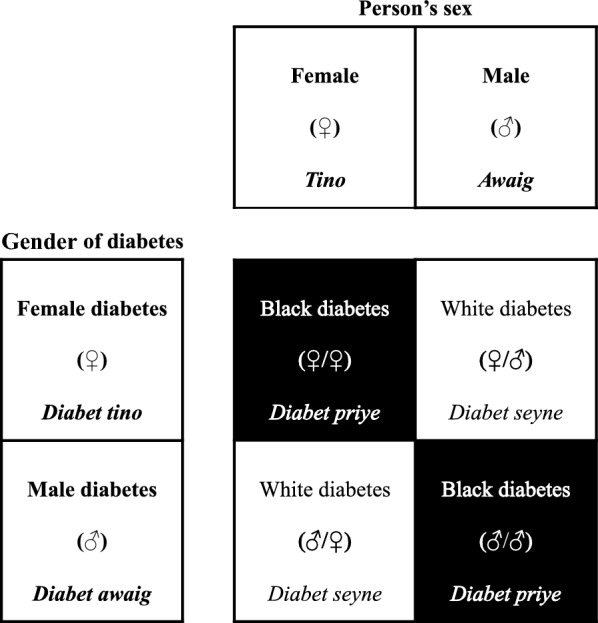
The interaction of diabetes types based on gender and colour according to the classification of Parikweneh from Macouria. The colour attributed to a person’s type of diabetes, namely white diabetes or black diabetes, represents the manifested interaction between a person’s sex and the underlying gender granted to the illness: i.e. male diabetes or female diabetes

According to the Parikweneh of Saint-Georges and Macouria, there were traditionally no names for diabetes in Parikwaki due to the relatively recent discovery of the disease. However, participants surmised that diabetes may have been around much longer than reported and mistakenly referred to as *imasewnti* in the past, an illness of shamanic origin sent through spells. Participants now recognize multiple forms of diabetes named according to various criteria of classification, two of which are intricately related (Fig. 2). These include:

- two forms based on biomedical nomenclature, i.e. type 1 diabetes (T1D) and T2D;
- three forms of diabetes based on colour, i.e. *diabet seyne* (white diabetes), *priye* (black) and *duweh* (red);
- two forms of diabetes based on gender, i.e. *diabet awaig* (male diabetes) and *tino* (female);
- two forms of diabetes based on age, i.e. *diabet himano*^1^ (young diabetes) and *kiyapye* (old).

#### Symptoms and classification

**Fig. 3:**
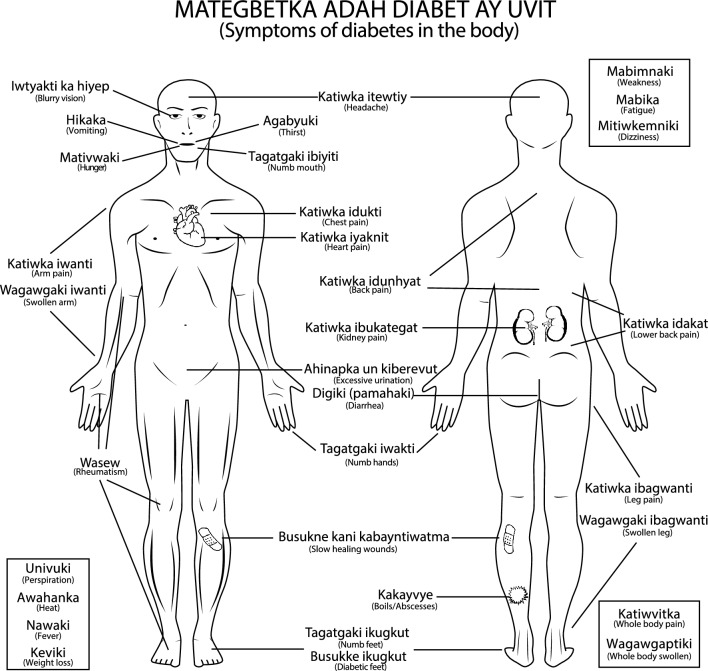
Symptoms, complications and problems associated with diabetes listed by Parikweneh participants from the communes of Saint-Georges and Macouria. Refer to Table 2 for details on symptoms and terms in Parikwaki

The current Parikweneh classification of diabetes is based on the observation of symptoms and complications encountered by participants with diabetes. First and foremost, 35 symptoms and complications associated with diabetes, which could be grouped into 15 overarching categories, were listed by Parikweneh participants (Table 2; Fig. 3). The 10 most cited symptoms were (1) weakness and fatigue, (2) weight loss, (3) troubled vision, (4) fever, (5) polyuria (i.e. excessive urination), (6) pain, (7) slow healing wounds, (8) polydipsia (i.e. excessive thirst), (9) diarrhoea and (10) increased hunger.

**Table 2:** Symptoms, complications and problems associated with diabetes listed by Parikweneh participants from the communes of Saint-Georges and Macouria

Rank	FC (%)^a^	English	Parikwaki	Description
1	93.6	Weakness	*Mabimnaki*	General feeling of weakness and lack of strength
8	55.3	Fatigue	* Mabika*	Chronic fatigue and feeling of tiredness
16	19.1	Dizziness	* Mitiwkemniki*	Feeling of faintness at the peak of weak spells
2	78.7	Weight loss	*Keviki*	Quick or sudden weight loss regardless of appetite. Often associated with severe cases of diabetes
3	72.3	Blurry vision	*Iwtyakti ka hiyep*	Literally “the eyes cannot see” from *iwtyakti* “eye”, *ka* “negation” and *hiyep* “to see”. Blurry vision can be temporary, coming and going with poor glycaemic control, or permanent, leading to blindness
4	68.1	Fever	*Nawaki*	Feeling of increased body heat, notably after bouts of hard work or weak spells
5	66.0	Excessive urination	*Ahinapka un kiberevut*	From *ahinapka* “to go to the washroom”, *un* “water” and *kiberevut* “often”; generally accompanies excessive drinking
6	63.8	Pain	*Katiwka*	Pain occurring throughout the body
13	25.5	Headache	*Katiwka itewtiy*	From *katiwka* “pain” and *itewtiy* “head”
17	17.0	Back pain	*Katiwka idunhyat*	From *katiwka* “pain” and *idunhyat* “back”
27	6.4	Lower back pain	*Katiwka idakat*	From *katiwka* “pain” and *idakat* “the lower back”; generally employed when referring to pain in the kidney area
34	2.1	Kidney pain	*Katiwka ibukategat*	From *katiwka* “pain” and *ibukategat* “kidney”; specifically employed when it is known that the kidneys are hurting, e.g. dialysis
35	2.1	Chest pain	*Katiwka idukti*	From *katiwka* “pain” and *idukti* “chest”; generally employed when pain is felt in the chest or heart area
31	4.3	Heart pain	*Katiwka iyaknit*	From *katiwka* “pain” and *iyaknit* “heart”; specifically employed when it is known that the heart is hurting
18	17.0	Leg pain	*Katiwka ibagwanti*	From *katiwka* “pain” and *ibagwanti* “leg”
28	6.4	Arm pain	*Katiwka iwanti*	From *katiwka* “pain” and *iwanti* “arm”
19	17.0	Whole body	*Katiwvitka*	Specifically when general pain is felt throughout the body
22	12.8	Rheumatism	*Wasew*	Specifically when pain is felt in the joints and articulations
7	63.8	Slow healing wound	*Busukne kani kabayntiwatma*	Literally “the wound is not well” from *busukne* “wound”, *kani* “negation” and *kabayntiwatma* “well”
21	17.0	Diabetic foot	*Busukke ikugkut*	Literally “foot sore/wound” from *busukke* “sore/wound” and *ikugkut* “foot”; this refers to the ulcers developing on the feet of diabetics
23	12.8	Boils/abscesses	*Kakayvye*	From *gakay* “pus”; this is the general term for any boil-like or abscess-like wound. There are different types like *kumeh*, *wahagu* and *wagewni*
9	53.2	Thirst	*Agabyuki*	Increased, excessive and continuous drinking without the feeling of hydration
10	51.1	Diarrhoea	*Digiki*	Reoccurring symptom; can accompany the consumption of a food item that does not agree with the disease. *Pamahaki* meaning “salty fish” may be used to be discrete
11	42.6	Hunger	*Mativwaki*	Increased, excessive and continuous eating without the feeling of satiety
12	31.9	Heat	*Awahanka*	Feeling of increased body heat, akin to fever, notably after bouts of hard work
14	21.3	Numbness	*Tagatgaki*	Feeling of numbness or discomfort occurring, namely in the extremity of limbs or in the mouth
25	8.5	Numb feet	*Tagatgaki ikugkut*	From *tagatgaki* “numbness” and *ikugkut* “foot”; of particular concern when injuries are implicated
30	6.4	Numb hands	*Tagatgaki iwakti*	From *tagatgaki* “numbness” and *iwakti* “hand”
24	10.6	Numb mouth	*Tagatgaki ibiyiti*	From *tagatgaki* “numbness” and *ibiyiti* “mouth”; occurring, namely after eating something not agreeing with the disease
15	21.3	Vomiting	*Hikaka*	Recurring vomiting particularly at the peak of heat and weak spells
20	17.0	Swelling/inflammation	*Wagawgaki*	Quick and lasting swelling and inflammation from bumps and bruises, as well as general swelling in the limbs
29	6.4	Swollen legs	*Wagawgaki ibagwanti*	From *wagawgaki* “inflammation” and *ibagwanti* “leg”
32	4.3	Swollen arms	*Wagawgaki iwanti*	From *wagawgaki* “inflammation” and *iwanti* “arm”
33	4.3	Whole body	*Wagawgaptiki*	Specifically when the whole body is inflamed and swollen
26	8.5	Perspiration	*Univuki*	Excessive perspiration particularly at the peak of heat and weak spells

^a^Relative frequency of citations (FC) expressed as a percentage

Although some Parikweneh participants could name the biomedically recognized forms of diabetes, namely type 1 and type 2 (14.6%; 7/48 interviews), the majority of those interviewed (72.9%; 35/48) identified various forms and classifications of the disease depending on how symptoms and complications appeared in an individual and the severity of their outcome (Fig. 2). The most commonly cited classificatory criterion, recognized in Saint-Georges and Macouria, was colour (60.4%; 29/48). Diabetes was primarily distinguished between its black form (*diabet priye*) and white form (*diabet seyne*), although some participants recognized a red form (*diabet duweh*). In all cases, white diabetes was the least severe, characterized by few complications and a slow progression of the disease, and this form is the easiest to manage and treat. Moreover, people reportedly have more dietary freedom, gain weight as expected and, in the best-case scenario, can be cured.

Black diabetes is more severe and deadlier than its white counterpart. It manifests itself suddenly with rapidly devolving symptoms such as polyuria, diarrhoea and vomiting that quickly lead to rapid weight loss and a dry emaciated look. It is difficult to treat and manage; without biomedical intervention from the hospital or dispensary, it may quickly lead to death. Furthermore, people with this form of diabetes can find their skin turning black. Although it is unheard or not recognized by most people, red diabetes is the most dangerous and deadliest according to some. Due to limited citations of this form, how it differed from that of black diabetes could not be clarified.

The second most cited classificatory system, described solely by participants from Macouria, was based on gender (27.1%; 13/48), whereby the illness was personified as being male (*diabet awaig*) or female (*diabet tino*) (Fig. 2). As LS6020 from Macouria explained*, “You see, you (a male individual) have the female diabetes, it*’*s because she thinks you*’*re her husband. That*’*s why she doesn*’*t kill you”*. In other words, male diabetes and female diabetes were considered incompatible with diabetic individuals of the same sex (i.e. men and women, respectively). Hence, the gender of the disease was determined by evaluating the severity of the illness in an individual through a logic of concordance of sexes and genders. Diabetics who respond poorly to treatments and are characterized by a considerable loss in energy and weight, as well as rapidly devolving health, are believed to have diabetes of the same gender. On the other hand, diabetic individuals who responded well to treatments and lived long and comfortably well with few dietary restrictions were believed to have diabetes of the opposite gender.

In comparison with the criteria of colour, the distinction of diabetes based on gender was not as concise, as their explanation was frequently intertwined with that of colour. One participant explained that male diabetes was in the bone and hard to treat, that female diabetes was in the blood and hard to treat, that white diabetes was in the skin and easy to treat, and that black diabetes and red diabetes were increasingly worse to treat than white diabetes. The most concise picture drawn by participants from Macouria was that the colour of a person’s diabetes was ultimately explained by gender (Fig. 2). Hence, the reason a person manifests a least severe form of diabetes (i.e. white diabetes) is because they are afflicted by a form of diabetes which itself is characterized by the opposite gender, whereas someone manifesting a more severe form (i.e. black diabetes) is the result of the opposite relationship.

Some participants (4.17%; 2/48), notably from Macouria, distinguished between a young form of diabetes (*diabet himano*) and an old form (*diabet kiyapye*)*,* although these could not be concisely described. In one interview, participants explained that old diabetes kills a person faster, whereas young diabetes does not, a statement contradicted by the reversed affirmation from other participants in another interview.

While most Parikweneh participants recalled having learned these terms from other members of their community, some specified having acquired these terms from doctors as well, although none of the healthcare staff interviewed could confirm this. Indeed, non-Indigenous participants affiliated with the healthcare sector were not familiar with the Parikweneh classification and description of diabetes. On the other hand, Parikweneh participants who cited T1D and T2D either stated that they were synonymous with black and white diabetes*,* respectively, or regarded the local classificatory system as something different in its own right.

#### Historic and current aetiology of the disease

Although the current Parikweneh classification of diabetes is based on observations of symptoms and complications, the evolving nomenclature of the disease reveals the most of its perceived origin and cause. Participants explained that diabetes probably existed in their communities prior to contemporary knowledge of this illness. Pointing to a series of illnesses and deaths in the 1950s in Kumene (Urucaua), in the Brazilian state of Amapá, nearly half of Parikweneh participants (47.9%; 23/48) surmised that these, mistakenly taken for another illness of shamanic origin called *imasewnti*, were likely some of the first cases of diabetes in their community. One participant from Macouria, LS6021, explained:

*“People used to say that it was the shaman who gave this spell. Like* imasewnti, *he made people who were big become skinny, dry, all that. Then they died. It was virtually false. That [happened] when he got diabetes. Diabetes, him, he acts so quickly that the person doesn’t realise that it’s really diabetes. He just eats and eats and eats. And then when he eats a lot, that’s it. He sees that he’s losing weight and then he loses his appetite, and all that.”*

Referring to a rack used for storing, cleaning or grilling food, *imasewnti* was ascribed to people afflicted by weakness and, more characteristically, sudden weight loss. Resulting in the loss of fluids through polyuria and diarrhoea in people exhibiting extreme thirst, as well as a darkening of the skin, *imasewnti* was believed to have dried out the afflicted akin to smoked or grilled flesh (i.e. meat and fish). Moreover, *imasewnti* was contracted through spells (*timnaka*)*,* malicious intent sent by a shaman (*ihamwi*) or a jealous acquaintance by means such as performing an incantation (*aviri*^2^) and blowing into a glass of water meant for ingestion by the recipient in question or blowing into wood burning for grilling or smoking food. As one Elder from Saint-Georges, LS5017, explained, “imasewnti *can mean that people know how to blow it. Like a wind that*’*s going to smoke. That*’*s who they call* imasewnti. *You become skinny, skinny, skinny; you become dry*”*.*

The increasing incidence of *imasewnti* and growing distrust is believed to have culminated in the late 1950s with the assassination of a shaman in 1959 at the hands of over a dozen family heads in retribution for his perceived implication in the death of another. Although this led to the capture and imprisonment of some implicated actors by Brazilian law enforcement, many others fled with their families, taking refuge on the French Guianese border. Indeed, an Elder recalled how her mother, pregnant with her at the time, fled to French Guiana after her father, who had been involved in the incident, left the family. Further corroborating this, Roger Labonté, the current chief of Espérance 1, estimates that eight families, fleeing repercussions, established themselves in French Guiana to later found the department’s first Parikweneh village in 1963, Espérance 1. Antonio Felicio, the past chief of Kamuyene, confirmed this incidence and added that more than 20 families subsequently settled on the French side of the Oyapock River in Ouanary, Trois-Palétuviers and Saint-Georges.

Parikweneh’s oral history subsequently tends to suggest various paths by which Parikweneh came to learn about diabetes in a biomedical sense. The most influential and predominant path appears to be attributed to the arrival of American evangelical missionaries, linguistic scholars and academic authorities of the Parikwaki language: Diana and Harold Green in the 1960s. Participants recall that during their missions in Parikweneh villages in Brazil, where they conducted extensive linguistic work, they witnessed the devastating effect of diabetes and counselled Parikweneh in seeking biomedical treatment for this disease. It is believed that their implication eventually contributed to discrediting the role of supernatural forces in this pathology, and the biomedical concept of diabetes was slowly integrated and subsequently exported to French Guiana in subsequent migration events.

Although at the time of the study, some Parikweneh participants believed that *imasewnti* still exists as a separate illness to diabetes, most associated its prevalence in their community with a dietary origin (Table 3). The most commonly cited causes and factors involved in the development of diabetes were the introduction and increased accessibility of sugar and sweet food items, namely sweet and carbonated beverages (i.e. juice and soft drinks), candies and treats and manufactured coffee and chocolate, thus highlighting their commercial provenance. When listing coffee and chocolate, which are typically consumed as breakfast beverages, participants pointed to the common practice of sweetening these beverages. Nonetheless, unsweetened and particularly homemade products derived from the coffee (*Coffea* spp.; Rubiaceae) and cacao plants (*Theobroma cacao* L.; Malvaceae), both cultivated by some Parikweneh, that retained a bitter taste were not thought to exacerbate diabetes, as bitterness is generally associated with health benefits.

**Table 3:** Relative frequency of citations (%) of the causes and factors responsible for the development of diabetes

Cause of diabetes	Frequency of citations (%)		
	Parikweneh (*n* = 48)	Healthcare (*n* = 29)	All^a^ (*n* = 75)
Sugar and sweet foods	100.00	93.10	97.33
Sugar	97.92	86.21	93.33
Sweet and carbonated beverages	66.67	51.72	61.33
Coffee (*Coffea* spp.)	37.50	6.90	25.33
Candies and treats	22.92	13.79	20.00
Chocolate (*Theobroma cacao*)	29.17	0.00	18.67
Starch and starchy foods	91.67	82.76	88.00
Cassava tubers (*Manihot esculenta*)	91.67	79.31	86.67
Rice (*Oryza sativa*)	20.83	41.38	28.00
Red beans (*Phaseolus vulgaris*)	12.50	27.59	17.33
Starch	6.25	37.93	18.67
Wheat-based pasta and bread (*Triticum* spp.)	10.42	10.34	10.67
Potatoes (*Solanum tuberosum*)	4.17	0.00	2.67
Fat and fatty foods	50.00	51.72	49.33
Fat	37.50	34.48	36.00
Oil	31.25	17.24	26.67
New recipes	8.33	17.24	10.67
Salt and salty foods	37.50	34.48	36.00
Salt	33.33	20.69	28.00
Salty foods	8.33	20.69	13.33
Alcohol	18.75	44.83	28.00
Beer	12.50	17.24	13.33
Cachiri (cassava beer)	8.33	3.45	6.67
Rum	4.17	3.45	4.00
Genetics	12.50	41.38	22.67
Weight control	8.33	44.83	22.67
Obesity and overweight	6.25	37.93	18.67
Lack of physical exercise	4.17	20.69	10.67
Industry produced and conserved foods	16.67	20.69	18.67
Chicken (*Gallus gallus domesticus*)	12.50	13.79	13.33
Beef (*Bos taurus*)	4.17	0.00	2.67
Coconut milk (*Cocos nucifera*)	2.08	0.00	1.33
Various new food items	22.92	0.00	14.67
Onions (*Allium cepa*)	14.58	0.00	9.33
Garlic (*Allium sativum*)	12.50	0.00	8.00
Spices	12.50	0.00	8.00
Pepper (*Piper nigrum*)	4.17	0.00	2.67
Pancreas failure	10.42	13.79	10.67
Insulin resistance and deficiency	10.42	13.79	10.67
Pancreatitis	2.08	3.45	1.33

^a^Two participants linked to the healthcare sector were also Parikweneh from Saint-Georges and Macouria

Starch and starchy foods were the second most commonly cited cause and factor involved in the development of diabetes. Although most Parikweneh participants drew a link between cassava tubers (*Manihot esculenta*) and its food derivatives (i.e. torrefied cassava semolina [*puveye*, or *kwak*^3^ in French Guianese Creole] [18], tapioca [*kayut*] and cassava juice [*karahu*]) and the development of diabetes (Table 3), they were quick to add that it was not the cause of the illness. More precisely, participants explained this to be the introduction of new food items such as rice (*Oryza sativa* L.; Poaceae), red beans (*Phaseolus vulgaris* L.; Fabaceae), wheat-based pasta and bread (*Triticum* spp.; Poaceae) and potatoes (*Solanum tuberosum* L.; Solanaceae), which increasingly accompany *kwak,* the staple food derivative of cassava roots. The link between *kwak* and diabetes was contentious, as participants frequently reported that healthcare professionals explained this to be the cause. The implications of this have been addressed in-depth in a previously published paper focussing on the role of cassava consumption in diabetes self-management strategies adopted by Parikweneh [18]. Briefly, however, the issue is presented here by participant LS5030 from Saint-Georges:

“Kwak *is the basis of our diet. We drink* kwak* in coffee. We dip mango in* kwak, *everything! A lot of doctors say diabetics can’t eat* kwak. *But if you’re protecting your body, you can eat* kwak*! [...] it’s important to have the cassava fields to be able to eat. But we’re told we can’t eat it or we’ll get diabetes. But if you don’t eat* kwak, *you don’t eat. But the doctors don’t understand that. What Palikur have is what they can eat. Palikur are obliged to eat* kwak. *It’s what we know how to grow! We can leave* kwak *aside maybe for a day, but after that, we want to eat it!*”

In fact, new food items to the current Parikweneh diet were the common denominators of most factors associated with the development of diabetes: from fat and fatty foods, often associated with new cooking practices requiring oil, to salt and salty foods (i.e. chips), to commercially processed foods and meats (i.e. frozen chicken [*Gallus gallus domesticus* (Linnaeus, 1758); Phasianidae] and beef [*Bos taurus* Linnaeus, 1758; Bovidae]), to easily accessible market foods such as onions (*Allium cepa* L.; Amaryllidaceae), garlic (*Allium sativum* L.; Amaryllidaceae), black pepper (*Piper nigrum* L.; Piperaceae) and spices, which are omnipresent in the local creole cuisine.

The causes and factors associated with the development of diabetes cited by Parikweneh participants were positively correlated with those cited by participants from the healthcare sector (*ρ* = 0.532, *p* < 0.001; Table 3). Nonetheless, the rankings of alcohol, genetics and weight control were some of the causes and factors that were markedly different between the two groups; these were some of the least frequently cited by Parikweneh participants. The biggest difference between both groups of participants was the topic of weight control, either through discussing obesity and overweight or the lack of physical exercise as risk factors in the development of diabetes. Whereas weight control was the factor that was the least cited by Parikweneh, it was discussed by nearly half of the participants linked with the healthcare sector (Table 3). Furthermore, in discussing genetics, Parikweneh participants questioned how this could be transmitted from one generation to the next and the role of pregnancy. Alternatively, some participants provided another description of the development of the illness. As LS5030 from Saint-Georges explains, diabetes, embodied by sugar, was already in the body; if you nourish it by eating poorly, it grows and manifests itself. This is supported by LS6020 from Macouria, who explains, *“When you*’*re born, you already have sugar in your blood. And as you grow up, if you don*’*t know how to control yourself with sweets, well, after that it becomes diabetes”.*

The contemporary name *diabet* is only supplanted in longevity by the name *sugku*, in reference to sugar, which is perceived as the predominant cause of diabetes. As LS6025 explains,

“Sugku*, it’s... it’s the same [as diabetes], actually. It’s when... For example, you go to the doctor to have your blood tested. Well... you can see sugar in the blood. Right... That’s it. And that’s what provokes it. That’s what provokes diabetes”.*

Hence, sweetness is a major component in the description of the disease. Nonetheless, some participants had a hard time consolidating sugar with diabetes due to the lack of access to it when it first appeared, as put forth by LS6027: *“Maybe in Brazil there is [sugar]… Macapá maybe. But in Urucaua, we didn*’*t have that. But how do Indians get diabetes if they don*’*t eat sugar?”﻿.*

### Knowledge of treatment

#### Biomedical treatments

In Parikwaki, biomedicine is called *nawohtunye giveykis*, which translates to “foreign medicine”. Overall, Parikweneh participants recognized that diabetes was treated through a number of its remedies. Pharmacotherapy through injections (81.2%; 39/48) and pills (70.8%; 34/48) were the most cited treatments (87.5%; 42/48). Most Parikweneh participants named insulin as a biomedical remedy for diabetes (62.5%; 30/48**)**, whereas only some named at least one antidiabetic pill, i.e. metformin, stagid, amaryl, velmetia or glibenclamide (8.33%; 4/48). Participants noted how these medications target blood sugar levels, such as insulin, and a number of comorbidities, such as high blood pressure and cholesterol.

Dietary interventions were cited by 83.3% (40/48) of Parikweneh participants who inevitably recognized the dietary origins of diabetes and the necessity of paying attention to diet along with pharmacotherapies. Although dietary recommendations cited by Parikweneh participants included increasing the consumption of vegetables such as tomatoes (*Solanum lycopersicum* L.; Solanaceae), cucumbers (*Cucumis sativus* L., Cucurbitaceae) and lettuce (*Lactuca sativa* L.; Asteraceae), the most cited practices related to food items are already well integrated into the Parikweneh diet. Haemodialysis was the least cited biomedical intervention (8.33%; 4/48). Although few participants discussed the benefits of physical exercise (16.7%; 8/48), it was difficult to determine if this was strictly perceived as a biomedical treatment. Whereas some explicitly stated that doctors advised diabetics to do physical exercise to manage that illness, it was also noted that this was important for maintaining good health in general, especially when reflecting on the lifestyle changes related to self-subsistence activities.

#### Parikweneh Treatments

Parikweneh call their medicine *Parikweneh giveykis*, which translates directly to “Parikweneh medicine” or “Peoples’ medicine”. Nearly all Parikweneh participants were able to cite at least one local remedy for diabetes (91.7%; 44/48) that targeted the control of blood sugar levels, other than biomedical treatments. Although these were solely plant-based, 89.6% (43/48) cited both animal and plant remedies against specific symptoms of diabetes (e.g. aches and pains, diarrhoea, polyuria and infected wounds).

#### Attitudes towards the illness

Parikweneh participants often associated diabetes with heat, whereby some diabetics experienced hot flashes and sometimes fevers. Symptoms such as headaches were compared to the head being hot, and the expression of certain symptoms such as inflammation, polydipsia and the evacuation of fluids through polyuria, diarrhoea and perspiration were linked to the body being hot and needing to be cooled by replenishing with water. Due to its association with sugar, sweetness is a recurring characteristic attributed to diabetes by Parikweneh participants. The notions that sugar and diabetes are in the blood were used interchangeably to describe glycaemic control, whereas hunger was linked to the illness being voracious for sugar. LS5012 goes as far to specify that sugar even finds itself in urine, having observed ants being attracted to it. These hot and sweet attributes of diabetes are important factors driving current attitudes towards this illness, particularly because the organoleptic and physical properties of Parikweneh remedies are often discussed. When discussing the bitterness of certain Parikweneh treatments, one participant, LS5007, drew a parallel with biomedical treatments: *“I always have the pills to drink. I drink, I drink, I drink. But… Because here, the pills and tablets… When you drink, it*’*s bitter, bitter, bitter, bitter in my mouth”.*

To make a case in point, a number of diabetic participants were willing to try and experiment with local remedies alongside biomedical treatments. Among diabetic individuals, 88.9% (24/27) were using biomedical treatments, 88.9% (24/27) named a Parikweneh treatment, whereas 88.9% (24/27) were using Parikweneh treatments (at the time of the study or in the past). Of the three who did not use Parikweneh medicines, two had used or tried them in the past. Only 11.1% (3/27) of the respondents reported using only local treatments. One person who did not use biomedical treatments reported no longer having diabetes, having healed from it with Parikweneh treatments. One diabetic respondent who used biomedical treatments reported that their spouse had also healed himself with Parikweneh treatments. Finally, one participant who only used Parikweneh treatments reported having never been to the doctor or receiving a diagnostic, having self-diagnosed herself by borrowing a glucometer.

In general, diabetic individuals (77.8%; 21/27) used both biomedical and local Parikweneh treatments, either at the same time or in alternation, to manage their diabetes. Despite an overwhelming lack of confidence in the healthcare sector and an apprehension about receiving biomedical treatments, diabetic participants recognized that there were too many barriers to relying solely on Parikweneh treatments. Acknowledging the seriousness of diabetes as an illness, diabetic individuals underscored the importance of obtaining a diagnosis when the first signs were detected and continuing medical consultations to obtain examination results, as well as regular use of glucometers. Hence, detection was performed directly at a doctor’s clinic or more conveniently in the village either when home nurses visited a household or by borrowing a glucometer from a family member or friend. These tools are essential for evaluating the success of Parikweneh treatments because biomedical treatments are sometimes excluded and not taken for months at a time; thus, they are replaced entirely by local medicines. These remedies were all prepared in the household by diabetics themselves or another member of their household. Although knowledge of medicinal practices and treatments remained largely familial, sharing of knowledge between households was not uncommon. Only one diabetic patient consulted a knowledge holder who had come to French Guiana from Brazil for that purpose.

### Practices

Practices regarding the management of diabetes revolve around a combination of dietary adaptations and surveillance, as well as locally practiced medicines and remedies. Due to the dietary nature of diabetes, Parikweneh participants observed the effect of a number of animal and plant species on glycaemic control and the management of diabetes (data not shown).

#### Dietary adaptations

Despite Parikweneh participants reporting dietary changes marked by a rise in the consumption of introduced store-bought foods, cassava roots continue to be the primary dietary staple in the Parikweneh food system. Due to the centrality of cassava in discussions, results pertaining to adaptations in the transformation of cassava roots for the dietary management of diabetes have been published separately [18]. Briefly, *kwak* (or *puveye* in Parikwaki) and cassava root were discussed by participants in 93.3% of all interviews [18]. However, Parikweneh participants (83.3%) reported two principal types of *kwak*, whereas this was only brought up by one non-Parikweneh participant working as a home nurse [18]. These were called sweet *kwak* (*puveye kiteye*) and acidic *kwak* (*puveye suweine*), whose names characterize their organoleptic properties, whereby most participants considered acidic *kwak* to be better for diabetic individuals [18].

Other dietary changes reported by diabetic participants were adopted on a personal basis. As demonstrated in the case of cassava consumption [18], these choices typically arise from negative reactions experienced after consuming specific food items that result in the following symptoms: (1) numbness in the mouth, (2) dry mouth, (3) sudden fatigue and weakness, (4) perspiration, (5) increase in body heat, (6) diarrhoea and (7) polyuria.

Blood glucose levels were at times assessed during these occasions, either by individuals themselves or with the assistance of a family member or nurse, to verify the presence of hyperglycaemia.

Among food items cited by participants for their adverse effect on diabetic individuals are included those originally listed in the development of diabetes (e.g. coffee [*Coffea* spp.]*,* chocolate [*Theobroma cacao*]*,* rice [*Oryza sativa*]*,* garlic [*Allium cepa*]*,*chicken [*Gallus gallus domesticus*]*,* swine [*Sus domesticus*; Suidae]*,* and beef [*Bos taurus*]; Table 3). Parikweneh participants also made similar observations regarding foods considered to be classically part of the traditional Parikweneh food system. These include nectars from the fruits of the patawa (*Oenocarpus bataua*)*,* bacaba (*Oenocarpus bacaba*) and açaí (*Euterpe oleracea*) palms (Arecacea), which participants linked to their high fat content, as well as several wild animals. In fact, participants were quick to generalize that white skin-coloured fish were compatible with diabetes whereas red fish with scales were not. A number of notably sweet fruits, locally grown or harvested, were also cited for their negative impact on glycaemic control, although participants noted that this did not apply to the consumption of unripe fruits like bananas (*Musa* × *paradisiaca* L.; Musaceae), mangoes (*Mangifera indica* L.; Anacardiaceae) and papayas (*Carica papaya*; Caricaceae). Finally, hot peppers (*Capsicum annuum* L.; Solanaceae) were advised against, especially when mixed with cassava juice as a condiment (*atit karahu*), with some suggesting that hot peppers should be avoided altogether when sick and undergoing intense treatments, biomedical or Parikweneh.

The key practices for determining which food items to avoid or consume included paying attention to body signals and monitoring glucometer readings. Adverse reactions and poor glycaemic readings have prompted some diabetic participants to identify specific foods that should be consumed in moderation or avoided altogether, whereas the opposite is true to identify which foods have a positive or neutral effect on diabetes. Nonetheless, participants stated overall that the reaction to food items was personal and could differ from one person to the next*,* as exemplified by LS6006 from Macouria when translating for his partner:

*“So in relation to the food she’s going to consume, so her mouth is going to... how shall I put this? Go numb. Yeah. It’ll go numb and... and the symptoms will appear. She’ll feel bad afterwards. So all that. Whether it’s... but mostly, it’s... fish. Fish from the sea. Or else... pig, other... Yeah, deer, all that. But it’s all about the person. Yeah, it’s each person. So she can eat... She could eat that and it goes with her. But there’s other people when they eat the same thing, there’ll be symptoms.”*

#### Animal and plant-based medicines and remedies

##### Species cited and used

Out of 48 interviews with Parikweneh people, participants were able to cite at least one plant used to treat diabetes in 44 interviews (data not shown)*.* The consumption of animals for the treatment of diabetes was cited in two interviews, although animals were more frequently cited for treating symptoms associated with the illness (data not shown). More precisely, the grease of animals is used alone or mixed with various plant oils to make creams and cataplasms to treat ailments such as muscle pains, arthritis, swelling and inflammation. Overall, animals and plants species were cited in 43 interviews for treating a number of symptoms associated with diabetes, such as polyuria, diarrhoea, abscesses and infected wounds.

Just as for food items, participants specified that the response to specific remedies targeting diabetes in particular was not the same for everyone. Once again, glucometer readings, taken individually or with assistance, were used to assess a remedy’s efficacy in the absence of symptoms. If a person experienced any of the adverse reactions listed for foods, this remedy was not deemed appropriate for that person.

Due to the sweet and hot characteristics attributed by Parikweneh to diabetes, Parikweneh treatments were frequently described based on their organoleptic (i.e. sour and bitter tasting) and caloric (i.e. cold, cool and refreshing) properties. This was particularly true for plant species. Furthermore, Parikweneh participants noted how many bitter tasting species cited against diabetes were used for other health problems that Parikweneh associated with heat, such as polyuria, fever and malaria.

##### Remedy administration

The remedies used internally included macerations, infusions or decoctions, and the remedies used externally included washes, tinctorial rubs, steam baths, cataplasms and creams. The remedies were either composed of a single species or a mixture. Although remedies can be prepared from fresh plant parts, the bark is often dried and stored for later use. Some methods of preparation also acted as preservation methods whereby creams and tinctorial extractions could also be stored. Unless deemed inefficient, plant remedies targeting diabetes were rarely treated as single-use cures for this illness and are seldom used only once. Instead, these were consumed for short or long periods of time, ranging from a week to a month, to daily use. In such cases where remedies are used for long periods of time, their posology, or dosage, is akin to biomedical remedies against diabetes particularly in terms of frequency: they are taken two to three times a day (i.e. morning, noon and evening). Alternatively, they may be consumed simply like water when one is thirsty. Remedies prepared for these uses are water-based, resulting from macerations, infusions or decoctions, and are generally stored frozen or refrigerated for later use.

#### Spiritual interventions

When recounting the history of diabetes in relation to shamanism, many Parikweneh participants added that shamans were no longer part of the Parikweneh medicinal system, having now been replaced by doctors or church pastors. When considering the remainder of Parikweneh participants, it remains clear that if Parikweneh shamans still exist, they are marginalized and live excised from the community, largely due to the effect and influence of evangelization [48, 49]. People who visit them do so discreetly. Although the preparation and use of Parikweneh medicines appear widespread, people are modest regarding their knowledge to not give off the wrong impression.

Not surprisingly, none of the diabetics interviewed reported having consulted a shaman for their diabetes. However, one elderly participant who had begun the initiation rites to become one in his youth had resorted to some of these practices for his diabetes. More precisely, a number of plants were used, not to target diabetes and glucose control per se but to harness the strength of the spiritual relationship he had long stopped cultivating with these plants. These included smoking ceremonies with tobacco (*aig*^4^ [*Nicotiana* tabacum; Solanaceae]) and prayers, as well as respect for strict preparatory guidelines and dietary restrictions.

To a certain extent, some participants did evoke the power of prayers in their remedies (27.1%; 13/48), whether for diabetes and its symptoms or for any other problem. These may have a Christian undertone due to the presence of, and the participation in, Evangelical churches in the communities, although some participants were quick to remind that prayers, chants and incantations were also traditional Parikweneh practices. According to LS5017 from Saint-Georges, “*For* kumeh *and* wagewni *(types of abscesses), they have its prayer. After the prayer, the remedies are applied, and it gets better. They*’*re Parikweneh prayers, the Elders knew that. Young people don*’*t know that anymore”.*

Despite this intergenerational gap in practices, knowledge of medicinal plants was primarily transmitted between individuals within the Parikweneh community. Knowledge from neighbouring populations (i.e. Indigenous, Creole, Brazilian) was also transmitted and integrated into new practices. Finally, three participants revealed an oneiric origin (i.e. relating to dreams) to some medicines used to treat diabetes, such as carambola (*Averrhoa carambola*; Oxalidaceae); two participants dreamed of their remedy themselves, whereas another dreamt of a Brazilian who then shared his knowledge. LS6015 explains how this was revealed in a dream:

*“But I can’t see the man! He was talking to me like this, he said: ‘Look at the plants.’ I said: ‘Ah, I know these.’ He said: ‘Well, take these! Wash... Wash yourself with this and take the roots. Drink it every day. Six months, he said, you’ll see. All the... All the sickness you’ve got, it’ll pass.’ Well, it’s true, isn’t it? I believed and then... I don’t know... You’re so traumatized by illness, I didn’t know that you had... effects on your dreams. How to put it?”*

## Discussion

### Knowledge

The history of diabetes among the Parikweneh provides a nearly impeccable reflection of the shifting worldview that marks their history. The historic representation of diabetes as the result of spells subscribes to documented Parikweneh conceptions of illnesses that result from supernatural forces [25, 36, 50], whereas its current links to dietary changes and blood glucose have taken a biomedical undertone. Furthermore, the notion of heat and cold that underlies the description of the disease and the effect of some of its remedies is similar to that apparent in the local Creole medicinal system [36, 51]. These notions have notably been traced back to neo-Hippocratic trends popular in European medical spheres in the sixteenth and seventeenth centuries opposing hot and cold in an alteration of Hippocrates’ theory of the four humours [36]. Although Descola [52] denotes an authentic Mesoamerican origin to hot–cold polarities and discredits an European inheritance as coincidence in Indigenous practices, he concedes that such forms of inclusive classification are typical traits of analogic ontologies. Whether this dichotomy regarding diabetes is indigenous to the Parikweneh is still under debate, but it represents their medicinal system in a light different from one historically described [25, 50] and ascribed to animistic or perspectivistic ontologies [36, 52, 53]. What is evident, however, is that the integration of diabetes into Parikweneh ethnomedicine appears to be one born out of incompatibility with exclusive animism but requires the coexistence of different ontological schemes. Such coexistence of animist and analogical ontologies has been described elsewhere, such as among Inuit in Canada by Laugrand [54], who explains the importation of the latter through the evangelization efforts of Christian Churches. The hybridization of ontologies therefore bears witness to historical events and the dynamic aspect of cultures, such as the nature of being, the representations of which are anything but fixed and immutable social facts.

In illustrating the role of *ihamwi* (i.e. shamans) in manipulating the supernatural forces that lead to disease, Grenand et al. [36] recollect the 1961 murder of a shaman, a story that bears a striking resemblance to the attributed catalyst of the discovery, or the acknowledgement, of diabetes reported here. Dreyfus [34] recollects a similar event from the late 1950s whereby a number of Parikweneh families took refuge in French Guiana to avoid persecution from Brazilian police and vengeful allies of the assassinated shaman. These events are likely one and the same to the one reported here in the aetiology of diabetes. Although participants estimated at least a decade or two before “diabetes” became a household name, this was done through the gradual acknowledgement that *imasewnti* was not the result of malice through the manipulation of supernatural forces but rather one related to diet. Indeed, by the end of the 1960s, shamans were still present on both sides of the Oyapock River along with the appearance of Evangelical Parikweneh pastors in Brazil [50].

The description of a dietary origin for diabetes among Parikweneh populations is reflective of a dietary shift already recorded in the literature. Such changes as the inclusion of sugarcane (*Saccharum officinarum*; Poaceae) and an increase in the consumption of cassava flour were reported in the late 1970s, during which hunting and fishing decreased at the expense of the agriculture of cassava, which supplied the city of Oiapoque [26]. Ouhoud-Renoux [30] links the increased reliance on cassava production for economic reasons to an increased reliance on cassava consumption at the expense of historically more diverse plant cultivation, exploitation and consumption. However, Vincent [29] reports rice and pasta, along with *kwak*, among the top three staple foods in Saint-Georges, attributing this competitiveness to a decreased production of *kwak*, its increased market value and a noted appropriation of biomedical discourse linking it to diabetes. Nonetheless, cassava was found to be a cultural keystone species that remained a staple food item relevant to Parikweneh, as evidenced by the important discussion around diabetes and current adaptations adopted for its production [18].

Despite these documented changes relating to Parikweneh subsistence activities and their link with the development of diabetes through dietary changes, there is an apparent general lack of discussion regarding the role of physical exercise and weight gain in these lifestyle changes. Indeed overweight and obesity were rarely mentioned by Parikweneh participants as a cause and factor involved in the development of diabetes, despite these being increasingly prevalent in French Guiana [55, 56] and biomedically recognized risk factors for the development of T2D [57, 58]. Why this is the case deserves further investigation. However, diabetes’ link to obesity and the importance of physical exercise is not lost on healthcare professionals. Here lies an area where important knowledge gaps may be bridged, but also, practically, for the development of lifestyle interventions and strategies that underscore, for example, how Parikweneh subsistence activities (e.g. hunting, fishing, wild-harvesting, and cassava farming) can increase physical activity to manage diabetes favourably.

Finally, the origin behind the Parikweneh classification of diabetes is ambiguous. Whereas some participants contend that these notions are Parikweneh in origin, others trace their various nomenclature to healthcare professionals. Although this could not be verified in this study, it is possible to hypothesize that this heterogeneous classification results from the consolidation of biomedical explanations provided by healthcare professionals and notions proper to the Parikweneh. As a result, old diabetes, young diabetes, black diabetes and white diabetes may be a tacit appropriation of the biomedical classification (type 1 vs. type 2) and an explanation for the disease’s evolution through time (prediabetes vs. diabetes). Indeed, insulin resistance is known to cause acanthosis nigricans [59], the darkening of the skin ascribed by participants to *diabet priye* (i.e. black diabetes). Such an observation, supported by biomedical explanations, is not incompatible with Parikweneh classificatory systems, which are accustomed to distinguishing similar plants and animal species based on the colour of their bark, skin or fur [36, 38, 60, 61]. Furthermore, the first reported cases of diabetes among the Parikweneh were published in 1978 after the author had been informed by community members of deaths following emaciation, polydipsia, and polyuria [26]. As some of the first symptoms historically associated with diabetes, it is not surprising that these are some of the most cited complications of the disease and have become defining characteristics of its different kinds.

As distant as the Parikweneh classificatory system of diabetes appears to be from the biomedical one, recent research suggests that a revised classification of diabetes may lead to more efficient and targeted treatments [62]. Based on six biological markers measured at diagnosis from 14,755 diabetic patients, Ahlqvist et al. [62] proposed redefining T1D and T2D into five novel subgroups characterized by the time of onset (early vs. late), body mass index (low vs. high), metabolic control (poor vs. moderate) and insulin production (deficient vs. resistant).

### Attitudes

Parikweneh People have shown demonstrable resilience to the appearance of diabetes amidst a rapidly changing way of life. This is demonstrated in attitudes towards the illness, which are strongly linked to change and foreign contact. This transpires through language, integrating the foreign name of the disease into Parikwaki and distinguishing the origin of medicines (i.e. foreign [*nawohtunye*] and Parikweneh). The same observation can be made with the Desana of the Upper Rio Negro in Brazil, who associated multiple infectious diseases, such as smallpox, measles and influenza, with European populations and given them names etymologically derived from Portuguese [63]. Despite its intrinsic and almost personified nature, lying in wait in an individual’s blood and luring them with the temptation of sugar, the Parikweneh very much view diabetes as an introduced disease through dietary and lifestyle changes that have increased through contact with non-Indigenous populations.

When a new disease is introduced, the knowledge system to which it is linked comes into contact with the local knowledge system. This can lead to mutual exclusion and cause the displacement of one system of knowledge and practices at the expense of the other [64–66]. However, it can also lead to medical pluralism, whereby both systems can coexist and even mix [44, 64, 67–69]. Similar to the Tsimane’ of the Bolivian Amazon [68], Parikweneh medicinal practices appear to coexist with biomedical practices as two discrete and separate entities, which is in line with observations made regarding the treatment of malaria [70, 71]. However, the contemporary Parikweneh concepts of diabetes appear to result from a process of syncretism with biomedical notions in a similar fashion to that observed with the integration of concepts on malaria in a Tanzanian community [67]. In other words, it appears evident that biomedical messages regarding diabetes are well understood, but their meanings are interpreted through Parikweneh concepts. Here, the creation of an authentic Parikweneh theory on the nature of diabetes is described.

The attributions of hot and sweet qualities to diabetes play an important role in shaping present attitudes towards the illness, acting as inadvertent bridges between biomedical and Parikweneh concepts. Sugar is associated with sweetness, the primary focus of diabetes treatments, with a diverse range of Parikweneh medicines distinguished by their organoleptic properties, such as acidity and bitterness. The symptoms connect the pathological explanations of diabetes to Parikweneh concepts of physical properties (i.e. heat, refreshing); hot symptoms are treated by numerous acidic and bitter plants. This translates into a large number of diabetic individuals using both biomedical and Parikweneh approaches in a systematic way that incorporates the acute awareness of signs related to hyperglycaemia and biomedical tools. Hence, biomedical remedies remain at arm’s length, whereas sensible observation of glycaemic control is used to assess the effect of home-grown dietary and therapeutic approaches with the ultimate goal of optimizing autonomy and reducing the reliance on pharmacotherapy.

However, proper glycaemic control is challenging for all diabetics, plain and simple. The ontological framework that allows for the convergence of biomedical concepts with Parikweneh concepts may begin with analogic concepts that overlap, but it may also end in a very different place, as illustrated by the attempt of LS5017 to employ the spiritual powers of specific plant species to treat his diabetes. In humanizing plants through the possession of a spirit and maintaining a relation of communication, this exhibits the typical markings of animism according to Descola [52] and Amazonian perspectivism described by de Castro [53]. Just as Descola [52] concedes that his ontologies are not mutually exclusive, our assessment of Parikweneh medicine highlights how one does not exclude the other. If therapeutic options fail to provide expected results, whether they be biomedical or Parikweneh, one may resort to the methods of the Elders.

### Practices

Although Parikweneh participants attributed the rising prevalence and incidence of diabetes in their communities mainly to the introduction of food items that are primarily market sourced, such as rice, wheat-derived foods, potatoes and red beans (Table 3), they did not refrain from observing the effects of indigenous or introduced food items on the management of diabetes in general. Vincent [29] notes a similar link between diabetes and purchased foods yet underscoring how traditional foods could not be the cause since no one used to be sick from diabetes in the past. Although this did not prevent participants from noticing their effect on glycaemic control, participants provided greater importance to market foods in causing the development of diabetes as opposed to foods that can be harvested or successfully cultivated at home or in the community. Despite these distinctions between *cause of* diabetes and *effect on* diabetes, restrictions on the consumption of cultivated hot peppers (*Capsicum annum*) and certain animals, such as red scaled fish, are noteworthy. *Capsicum annum*, for example, also appears in dietary restrictions that the Chayahuita of the Peruvian Amazon award to the treatment of leishmaniasis [72]. These dietary restrictions, or distinctions of positive and negative foods on the management of diabetes, also resemble notions of food strength and the *reima* system, where foods are classified as dangerous or inoffensive, which are documented throughout various societies of the Brazilian Amazon [73–75]. Perhaps the practice of observing the effect of foods on diabetes may stem from authentically ancient practices and beliefs on dietary restrictions regarding illness due to shamanic spells, common to Peoples of the Amazon, who may see themselves legitimized by the biomedical system, which also provides dietary restrictions in the management of diabetes.

From a biomedical perspective, starchy food items derived from rice, wheat, potatoes and cassava are characterized by medium to high glycaemic indices (GI). Because the calculation of insulin doses largely depends on carbohydrate consumption [76–78], GIs are helpful tools for making dietary choices, as replacing high-GI food items with low-GI foods improves glycaemic control [76]. Foods that are low in carbohydrates but high in proteins and fats, such as animal products, are therefore considered to have a low GI [79], with the added benefit that dietary fats have been found to delay gastric emptying, thus leading to a delayed rise in blood glucose [80, 81]. However, plasma free fatty acids have also been found to increase insulin resistance in muscles and liver, and to enhance hepatic glucose production in individuals with T2D [82–84]. In T1D, meals with carbohydrates and a high fat content have been found to cause sustained late postprandial hyperglycaemia [85], although Wolpert et al. [78] found great individual variability in the effect of dietary fat on insulin requirements, which adds credence to the Parikweneh perspective that people’s reaction to food varies from one person to the next.

In the contemporary Parikweneh context, many dietary and medicinal practices are explained through the scope of organoleptic dichotomies. Because most Parikweneh participants associated the development of diabetes with an increased consumption of sugar in foods and beverages, such observations strengthen the position of sweetness against acidic and bitter flavours as a central tenet of Parikweneh knowledge, attitudes and medicinal practices regarding diabetes. The sour canes (*Costus* spp.), which have been reported to be effective against diabetes by Parikweneh [36], are used a refresher due to their acidity. This duality appears to extend to bitter plants, as a number of plant species have historically been cited by the Parikweneh against diabetes (i.e. açaí [*Euterpe oleracea*]*,* quassia [*Quassia amara*]*,* *gongo* [*Geissospermum* spp.], caskfruit lianas [*Doliocarpus* spp.; Dilleniaceae], *tuu kamwi* [*Picrolemma sprucei*; Simaroubaceae]*,* *kwevan* [*Simaba orinocensis*; Simaroubaceae]*,* coffee senna [*Senna occidentalis*; Fabaceae]*,* guanandi [*Calophyllum brasiliense*; Calophyllaceae]*,* dragonsblood tree [*Pterocarpus officinalis*; Fabaceae]*,* cat's-whiskers [*Orthosiphon aristatus*; Lamiaceae]*,* yellow poui [*Handroanthus serratifolius*; Bignoniaceae] and *Aloe vera* [Asphodelaceae] [36].

The importance of organoleptic properties as a medicinal tenet is not unique to Parikweneh, as it appears relatively common throughout Amazonian societies and the Americas at large [86–90]. For example, Matsigenka considered bitterness and sourness as sensory qualities that are unpleasant to illness [87]. Although the organoleptic characteristics and classifications of plants arguably remain the result of sociocultural and personal interpretations [87, 91], taste perception is fundamentally biological. Bitterness, for example, is mediated in mammals by several G protein-coupled taste 2 receptors, such as those encoded by the TAS2R gene family present in humans, chimpanzees and bonobos [89, 92, 93]. Hence, taste can more appropriately be described as a complex biocultural process [87], one that is fundamental to the Parikweneh medicinal framework for the selection of biologically active plants based on bitterness. Yet, as compelling as it is to attempt to explain how the development of each new antidiabetic practice comes to be, it is, in reality, not possible for each case. The emergence of de novo uses may simply be invented [44, 94] or even have oneiric origins. This is exemplified here by the use of carambola (*Averrhoa carambola*)*,* which has been demonstrated to have in vitro and in vivo hypoglycaemic and antidiabetic effects [95].

## Conclusion

In the first published reports of diabetes among Parikweneh, Vieira-Filho [26] warned against acculturation and dietary changes on the health status of Brazil’s Indigenous people and advocates for greater consumption of traditional foods issued from hunting and fishing, as well as increasing physical activity. Indeed, the lasting impact of diabetes can be compared to that of the introduction of infectious diseases such as smallpox, measles and influenza. Certainly not in the number of lives taken, but in the impact, it has left and continues to leave on Parikweneh history, knowledge system and way of life. Just as infectious diseases have been integrated into Desana myths [63], diabetes has woven itself into contemporary Parikweneh oral history as a key component in explaining the displacement of shamanism and their migration to their current communities. Attitudes around diabetes show us that biomedical knowledge of the disease has not only been integrated into the Parikweneh knowledge system but also merged with authentic Parikweneh concepts. This translates into an array of home-grown practices using biomedical tools such as glucometers to guide dietary choices, phytotherapies and zootherapies. This points to a strong desire to autonomously manage diabetes. Although at-home visits from nurses are important in this process of self-management of diabetes, the lack of information regarding the nutritive qualities and antidiabetic potential of several plants and animals available to Parikweneh means that diabetic patients and their healthcare professionals must rely strongly on these self-assessment measures. Although such information is important in developing culturally adapted healthcare services, achieving this will require navigating the learning curb associated with applying the Nagoya Protocol. Currently, its application into French Law has led to several administrative (e.g. protocols and procedures), practical (e.g. identifying which populations or communities are concerned) and ethical (e.g. addressing how to consider genetic resources shared between peoples included and excluded by the protocol) challenges [47]. These issues will likely continue to hinder ethnobiological research in France and its overseas regions until they are adequately addressed. In the absence of readily accessible and adequate information, patient education programmes, such as those provided to individuals with human immunodeficiency virus (HIV) in Saint-Georges [96, 97], can be devised to leverage people’s motivation to self-management.

Messages related to diet and the implications of sugar and fat, to a lesser extent, appear to be well heard. However, messages about physical exercise and energy expenditure do not appear to be reinforced as much. This may explain why Parikweneh participants did not mention the role of obesity in the development of T2D [57, 58]. Linking energy expenditure to culturally relevant activities such as fallow-field cultivation, hunting, fishing, and the harvesting of natural resources for material and economic purposes (i.e. traditional roundwood construction [38]) may be helpful in making such recommendations accessible. This has the added benefit of acknowledging the value of local customs and practices while increasing access to diverse wild-harvested foods that play into the hand of food security. In the meantime, an in-depth look into the use of Parikweneh *materia medica* through ethnobiological investigations of diabetes and a range of health issues that go hand-in-hand with this illness, such as high blood pressure and cardiovascular diseases, may provide additional information about its possible properties. For example, the use of the syndromic importance value (SIV), developed by Leduc et al. [98], which focuses on symptoms and complications, may very well help to distinguish several potentially antidiabetic plant species that may not necessarily be cited when asked to list therapies against diabetes specifically.

## Data Availability

The dataset supporting the conclusions of this article is available from the Nakala repository (10.34847/nkl.e99ea8tv).

## Abbreviations

CDPS: Delocalized Centers for Prevention and Care (from French: *Centres Délocalisés de Prévention et de Soins*),
CHC: Cayenne Hospital Center Andrée Rosemon (from French: *Centre Hospitalier de Cayenne Andrée Rosemon*)
GI: Glycaemic index
KAP: Knowledge, attitudes and practices study
T1D: Type 1 diabetes
T2D: Type 2 diabetes

## Footnotes

1 Green et al. [42] translate the word *himano* as “girl” or “young girl”.

2 Green et al. [42] translate the word *aviri* as “spell” or “sorcery”.

3 Instead of the Parikwaki term *puveye*, the term *kwak* in French Guianese Creole is primarily used here as it is commonly used by Parikweneh when they are not expressing themselves in Parikwaki (highlighted by the verbatims used herein).

4 The Parikwaki spelling for tobacco varies between Grenand et al. [36, 37] (*aig*) and Green et al. [42] (*ayg*).
